# ANGIOTENSIN (1-7) EXPRESSING LACTOBACILLUS DOSE-DEPENDENTLY BENEFITS THE GUT-BRAIN AXIS IN AGED RATS

**DOI:** 10.1093/geroni/igz038.3109

**Published:** 2019-11-08

**Authors:** Yi Sun, Qiuhong Li, Amrisha Verma, Christy Carter, Thomas W Buford

**Affiliations:** 1 University of Alabama at Birmingham, Birmingham, Alabama, United States; 2 University of Florida, Gainesville, Florida, United States

## Abstract

Aging is associated with gut dysbiosis – a condition linked with altered central nervous system function (i.e the “gut-brain axis”). Age-related health benefits have been ascribed to the renin-angiotensin system (RAS), mediated partially via the angiotensin (1-7) or Ang(1-7) axis. This pre-clinical study explored dosing of a genetically modified probiotic expressing Ang(1-7) – which we previously showed to induce dose-dependent increases in circulating Ang(1-7) – in modulating the gut-brain axis. Twenty-nine male F344BN rats were randomized at 24 months of age to receive oral gavage of Ang(1-7) Lactobacillus paracasei (LP) zero (control), one, three, or seven times/week over 28 days. At day 29, samples of feces, serum and pre-frontal cortex (PFC) were collected. Microbiome taxonomic analysis of fecal samples was performed via 16S-based PCR. Serum samples were analyzed for tryptophan and downstream metabolites via LC-MS. PFC was evaluated for mRNA expression of select inflammatory cytokines. PCoA revealed that groups differed in the overall fecal microbiota community structure as determined by Unweighted UniFrac. Indices of alpha-diversity, including richness and phylogenetic diversity, displayed significant group differences – with the most dramatic effects observed in the 3-times/week group. Compared to control, serum serotonin and 2-Picolinic Acid were significantly increased in the 3-times/week group. The 3-times/week regimen also significantly reduced COX2, IL1β, and TNFα mRNA expression, and 7-times/week reduced COX2 and IL1β expression in PFC. Therefore, we conclude that short-term treatment with Ang(1-7) LP dose-dependently benefits the gut-brain axis in aged rats, with 3-times/week appearing to be the optimal dosing regimen.

